# Bridging length scales from molecules to the whole organism by cryoCLEM and cryoET

**DOI:** 10.1039/d2fd00081d

**Published:** 2022-08-12

**Authors:** Charlie Alexander Lovatt, Conny Leistner, René A. W. Frank

**Affiliations:** a Astbury Centre of Structural Molecular Biology, School of Biomedical Sciences, Faculty of Biological Sciences, University of Leeds LS2 9JT UK r.frank@leeds.ac.uk

## Abstract

Resolving atomic structures of isolated proteins has uncovered mechanisms and fundamental processes in biology. However, many functions can only be tested in the context of intact cells and tissues that are many orders of magnitude larger than the macromolecules on which they depend. Therefore, methods that interrogate macromolecular structure *in situ* provide a means of directly relating structure to function across length scales. Here, we developed several workflows using cryogenic correlated light and electron microscopy (cryoCLEM) and electron tomography (cryoET) that can bridge this gap to reveal the molecular infrastructure that underlies higher order functions within cells and tissues. We also describe experimental design considerations, including cryoCLEM labelling, sample preparation, and quality control, for determining the *in situ* molecular architectures within native, hydrated cells and tissues.

## Abstract 2

Experimental workflows combining mouse genetics, cryogenic correlated light and electron microscopy and cryo-electron tomography to bridge length scales from the whole organism to molecules.

## Graphical abstract

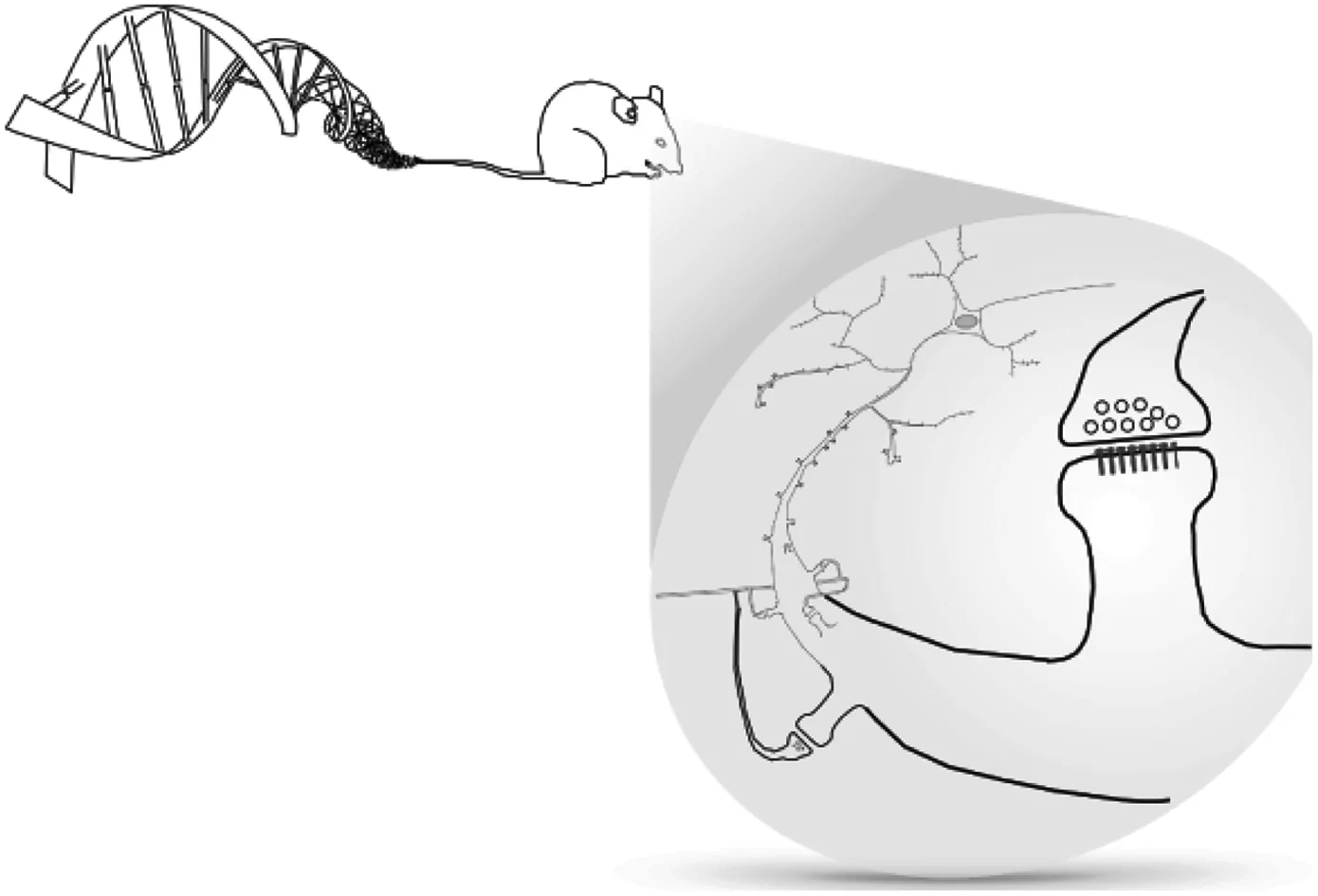

## Introduction

### Length scales of life

Mechanisms in biology can be appreciated at multiple distinct length scales, which poses a challenge for relating structure to function. For example, in the field of neuroscience, individual mice are used to investigate behavioural functions. However, the structures in the organism responsible for these functions span 9 orders of magnitude. Firstly, at anatomical subdivisions of the nervous system (centimetres to millimetres), the brain is composed of distinct subregions. For example, the hippocampal formation is essential for encoding episodic memories.^1^ Next, at the cellular level (millimetres to microns), neurons are organised within circuits.^2,3^ Each neuron is ordered into highly specialized subcellular compartments (microns to nanometres). For example, synapses are thought to be the primary loci for information encoding and storage within the brain.^4^ Lastly, each synapse is composed of several thousand macromolecular assemblies (nanometres to Ångstroms). In principle, a higher order biological function, such as behaviour, could be directly traced to ensembles of individual molecules, subcellular structures, and cells within a particular subregion of the brain. Therefore, a major challenge in neuroscience is to understand fundamental molecular mechanisms and associated diseases using methods that directly bridge length scales from molecules to the whole organism. More broadly, we suggest this challenge is applicable to many of the major questions within multiple biological and biomedical disciplines.

### Imaging molecules to whole organisms

Our current understanding of biological structure has arisen from a range of imaging modalities, each restricted to a particular biological length scale. Light microscopy is ideally suited to observe structures larger than ∼200 nm that are enhanced by optical sectioning to remove out of focus light.^5^ The low contrast and inability to resolve individual macromolecules directly necessitates the use of fluorescent labels in the form of small molecules, fluorescent antibodies, or fluorescent protein tags that are genetically expressed.^6^ Samples for fluorescence microscopy most often take the form of monolayers of chemically fixed cells or thin tissue sections. Imaging of viable tissues and animals is more difficult because at depths greater than a few hundred microns, the image is degraded by scattering and photodamage.^7–9^

Fluorescence imaging methods have the key advantages of both being able to target 1–3 specific proteins at a time with high-fidelity, and of having the potential to observe in the temporal domain to decipher dynamic processes in live specimens. On the other hand, the relatively small number of different molecules that can be simultaneously detected limits the breadth of structural information. For example, fluorescence microscopy is largely blind to the context of the proteins that are detected, including other macromolecular complexes, membranes and cellular compartments in which the fluorophores are situated.

Electron microscopy (EM) methods bypass some of these limitations. Despite the inherent low contrast of biological samples, label-free EM imaging with a ∼0.05 nm diffraction-limited resolution limit can reveal the structure of individual macromolecular complexes. The information obtainable by EM is critically dependent on whether or not samples remain in a fresh, native state for cryo-electron microscopy (cryoEM) or are fixed for conventional EM. Using the latter, fixation with chemical cross-linkers, organic solvents, and heavy metal stains increases the contrast of the sample and greatly simplifies image collection, particularly of thin tissue sections. However, this is at the cost of shrinkage, denaturation, or washing away of protein that prevents observing macromolecular complexes and membrane-bound organelles in their native state. Despite these limitations, tissue samples imaged by conventional EM resolve an ultrastructure of cell membranes and some organelles.^10^ Volumetric reconstruction by stitching together conventional EM images of serial sections or block-face imaging can identify the morphology and connectivity across tissues and of whole organisms.^11^

For cryoEM, advances in instrumentation, detectors and image processing have made single particle analysis (SPA) the most straightforward method for obtaining the native atomic resolution structure of macromolecules.^12,13^ Typically, electron micrographs are collected from purified proteins vitrified at cryogenic temperatures onto an EM grid.^14^ In general, a large image dataset of at least ∼10 000 randomly oriented, compositionally and conformationally homogeneous particles is required to obtain atomic or near-atomic resolution.^15^ However, as a consequence of the rigour of purification required to prepare samples for SPA cryoEM, proteins are not necessarily preserved in their native state and care must be taken to consider to what extent purification could have modified the native structure of a protein.^16^ Indeed, the structure adopted for some proteins may be unavoidably altered because SPA samples are prepared outside of the cellular or tissue environment.^17,18^

Built on the back of advances in SPA cryoEM, a new revolution is under way, in which structural biology is able to resolve macromolecular assemblies from inside cells and tissues by cryo-electron tomography (cryoET).^19^ For cryoET, multiple images of the target are collected as it is incrementally tilted between −60 and +60°, providing distinct views of the same object.^20^ Each view in the tilt series is a 2D projection of the 3D object. Thus, following alignment, the tilt series is computationally back-projected to generate a 3D tomographic density map.

Since samples are cryo-preserved in a hydrated, native state, in principle, near-atomic resolution structures are obtainable with cryoET data. This approach is particularly useful for pleomorphic samples, including reconstituted macromolecular assemblies and viruses that are otherwise not suitable for SPA.^21,22^ When collecting cryoET of cell and tissue samples, the raw tomographic map provides a 3D ‘molecular landscape’ composed of all macromolecules in the sample, with information to 2 nm resolution,^23^ which is sufficient to identify individual ∼250 kDa macromolecular complexes.^24,25^ Individual proteins much smaller than this can also be identified, particularly if the concentration of the protein of interest is high, the local background concentration of other macromolecules is low, or if the protein assembles into symmetric polymers, for example, cytoskeletal elements. If enough copies of a target protein are identifiable within a tomographic dataset, subvolumes containing the particle of interest can be aligned and averaged to obtain structures with resolutions comparable to those obtained by SPA cryoEM.^26,27^ In practice, the resolution achievable by subtomogram averaging is highly target and context dependent, wherein the intricate crowded environment of cells and tissues poses a particular challenge, with low copy number, heterogeneity of target molecules, and the presence of paralogues often the limiting factors. For small proteins in a crowded environment of a cell, assigning a protein’s identity within a raw tomographic map is thus a significant labelling problem for cryoET of cells and tissues.

A particularly exciting correlative imaging approach harnesses the labelling fidelity of fluorescence microscopy with the resolution and information richness of cryoEM by cryogenic correlated light and electron microscopy (cryoCLEM).^24,28^ The key advantage of cryoCLEM is in bridging length scales to be able to target the collection of cryoET datasets of specific proteins. This presents a solution for identifying specific, even rare, molecular structures within the vast and intricate molecular landscapes of cells and tissues. Cryogenic fluorescence microscopy (cryoFM) ensures the efficient and accurate targeting of cryoEM collections.^29,30^ In turn, cryoEM provides the *in situ* structure of target proteins and its context, including the native 3D molecular architecture in which the tagged protein resides.

## Experimental design

The precision of cryoCLEM is dictated by the accuracy of alignment of the cryoFM and cryoEM image, which is achieved using multiple fiducial markers identifiable in both the cryoFM and cryoEM images.^31^ The air objectives used for cryoFM (with a typical ∼0.9 numerical aperture) give a 50–200 nm positional accuracy,^28^ which is much less than the field of view of the cryo-tomogram (∼1.3 μm^2^). This is therefore sufficient to ensure the correct mapping of locations on the EM grid for cryoET data collection of the fluorescent target.^32,33^

**Fig. 1:**
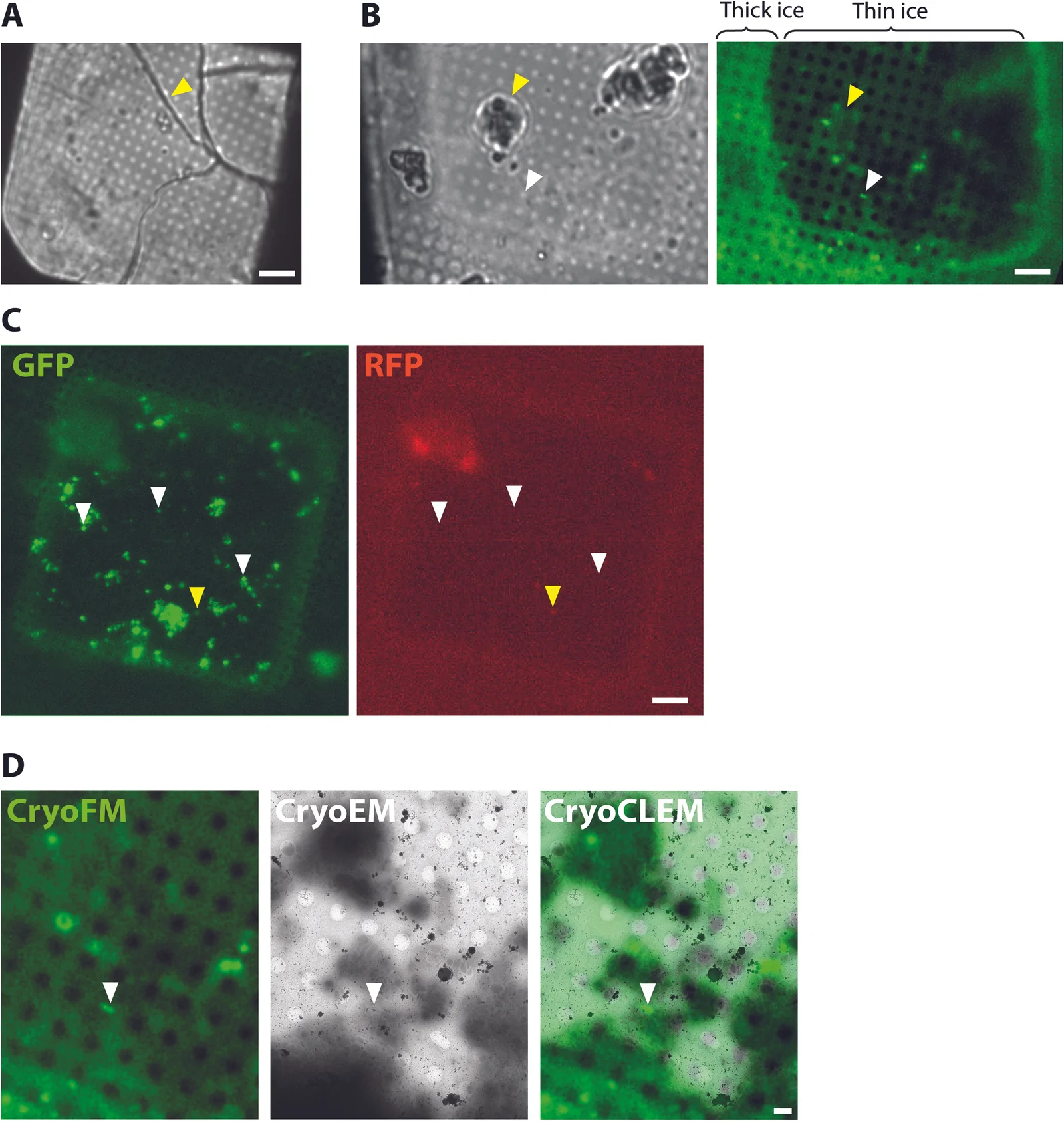
CryoCLEM of plunge-frozen synaptosome samples prepared from Psd95^GFP/GFP^ knockin mouse forebrain. (A and B) Using cryoFM to gauge ice thickness on EM grids. (A) Brightfield image showing the EM grid square. Yellow arrowhead, ice cracks indicative of non-vitreous sample. Scalebar, 6 μm. (B) Grid square with regions of thin ice. Left and right panels correspond to brightfield and GFP fluorescence channels, respectively. Regions of the grid square with thick ice are evident from increased autofluorescence of the holey carbon foil. White and yellow arrowheads, fluorescence region of interest within a hole of the carbon foil suitable for cryoET and surface ice contamination, respectively. Scalebar, 6 μm. (C) Detection of non-specific cryoFM signals by fluorescence in multiple different channels. Left and right panels, GFP and RFP channels, respectively. White and yellow arrowheads, GFP only puncta and non-specific autofluorescence or plastic contaminant, respectively. Scalebar, 6 μm. (D) CryoCLEM mapping of PSD95-GFP. Left, middle and right panels, GFP fluorescence channel, medium magnification montage electron micrograph, and merged channels, respectively. Images were aligned using holes evident from autofluorescence of the carbon foil as fiducial markers. White arrowhead indicates PSD95-GFP puncta mapped from cryoFM to the cryoEM image. Scalebar, 1 μm.

An important experimental design consideration in cryoCLEM experiments is the availability and type of fluorescent label, which is not trivial because this must be compatible with maintaining the viability of cells or tissues in an otherwise functional or physiologically ‘normal’ state. Thus, depending on the location of the target, biochemical or immunological labels must overcome physico-chemical barriers, including slow diffusion rates within tissues, lipid membranes and the crowded environment of cell cytoplasm.^34^ We therefore suggest that, in general, any labelling strategy that permits live imaging of the specimen should be applicable for a cryoCLEM experiment. For this purpose, genetically encoded fluorescent labels targeting the endogenous gene encoding a protein of interest have the advantage that stoichiometric labelling of every copy of the target is achieved with physiological levels of expression.^35–37^ For example, we have used knockin mice with fluorescent proteins targeted in-frame at the c-terminus of proteins (Fig. 1). Untagged wildtype mice serve as an ideal negative control to test the specificity of detection.^24^ It is also critical to consider functional assays that fully characterize genetic modifications to ensure that the introduction of the label has not perturbed functions relevant to the protein that has been tagged. For example, PSD95-GFP tags show no deficits, whereas homozygous PSD95-mEos causes synaptic deficits.^38^

However, as with live imaging experiments, genetic fluorescent tags targeted to endogenous genes are at the mercy of the copy number of endogenous expression. If the target protein is not concentrated within particular subcellular compartments or if the copy number is relatively low, one runs the risk that the fluorescent tag may be below the threshold of detection. While over-expression of a fluorescent fusion protein could mitigate the low copy number and give exciting insights,^39^ in some experimental settings this may give rise to architectures that deviate from physiological relevance.

Applying these experimental design considerations, we have developed two workflows combining mouse genetics, cryoCLEM, and cryoET to determine molecular architectures of target molecules within cells and tissue.

## Results

To collect cryoCLEM data we first imaged EM grids by cryoFM and assessed the quality of the sample, including sample thickness and the presence of any large crystalline ice contaminants. Non-vitreous samples were identifiable by the appearance of cracks observed in brightfield images. As shown in Fig. 1, thinner regions on the grid most suitable for cryoEM were also identifiable by the lower background autofluorescence. The specificity of detection was assessed with control samples and the collection of images from additional fluorescent channels.^40^ Autofluorescent false positive signals, including microplastic particles and ice contaminants, were apparent as puncta present in multiple fluorescent channels (Fig. 1). Samples of sufficient quality were then imaged on a 300 keV Titan Krios. The initial correlation of cryoFM and EM images was estimated by manual alignment. Fiducial markers, including the carbon foil on the EM grid or tetraspeck beads (Thermo Fisher) added to the sample, were mapped from the cryoFM images onto medium magnification montage EM images,^41^ to identify where fluorescent targets were located within the EM images (Fig. 1). Following cryoET data collection, the alignment of cryoFM and cryoEM images was repeated computationally with ∼10 fiducial markers to ensure a higher accuracy.^28^

### CryoCLEM of cells

**Fig. 2:**
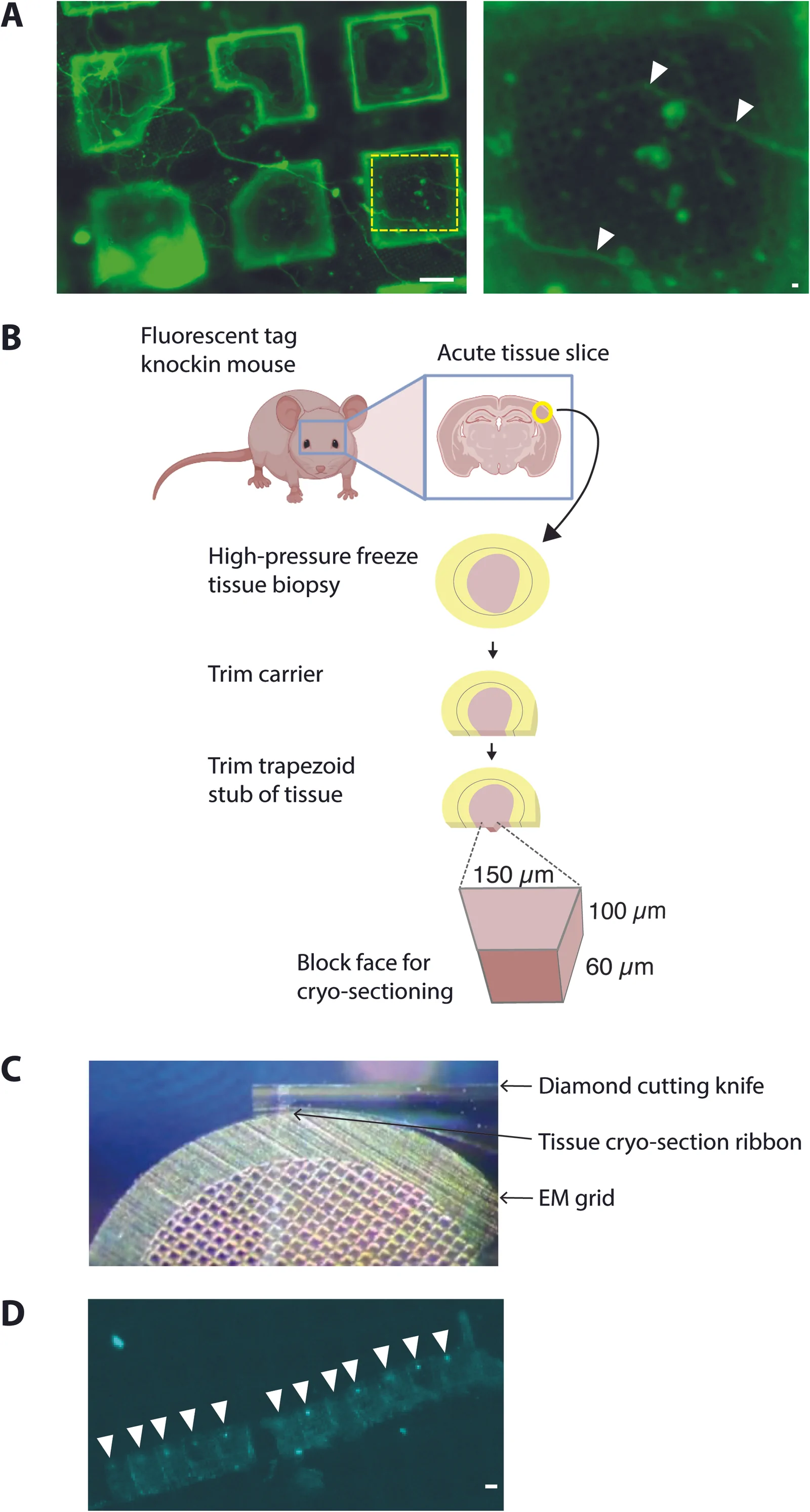
Sample preparation of cells and tissues on EM grids. (A) Dorsal root ganglion primary neurons grown on the EM grid and incubated with Alexa fluor 488-conjugated isolectin-B4 before plunge-freezing. Left panel, neurites grew across multiple grid squares. Scalebar, 20 μm. Yellow hatched box shown enlarged in right panel. White arrowheads, thin neurites traversing holes in the carbon foil. Scalebar, 1 μm. (B) Schematic indicating the workflow for preparing fresh adult mammalian brain for cryo-sections from high pressure-frozen brain tissue. Mouse brains were dissected and 100 μm thick acute slices were collected on a vibratome. 2 mm diameter biopsy samples were transferred to gold carriers and high-pressure frozen. Carriers were trimmed to prepare a trapezoid block of tissue, from which 150 nm thick cryo-sections were collected. (C) Image showing transfer of 150 nm thick cryo-section tissue ribbon from the diamond cutting knife to an EM grid. (D) CryoFM image of mouse brain cryo-section tissue ribbon. White arrowheads, individual cryo-sections within the tissue ribbon. Scalebar, 20 μm.

One of the prime considerations when preparing cells and tissues for cryoET was minimizing deviation from the physiologically normal state. A straightforward method was to culture cells *in vitro* directly on EM grids (using carbon and gold supports), followed by plunge freezing (Fig. 2). Cells grown on EM grids adhered to the carbon foil, and growth was enhanced by coating grids with poly-lysine and laminin. Critically, the regions of interest in the cell must be less than 500 nm thick, which represents the current upper limit for resolving macromolecules in cells by cryoEM. Some cell types were therefore more amenable to this approach^42^ such as primary neuronal cultures that grow distal subcellular processes, including neurites and synapses, many of which are thin enough for cryoET.^43,44^ However, cells cultured *in vitro* tended to grow to fit within the available space on the EM grid. For example, axons and synapses often avoided the holes in the carbon foil (Fig. 2), limiting the number of locations suitable for cryoEM. Neurites crossing holes typically grow to fill the available space in the hole. Consequently, the molecular architectures obtained from cells grown on cryoEM grids could be, at least in part, determined by non-physiological factors, including interactions with the holey carbon foil on the EM grid.

A further potential drawback of *in vitro* cultured mammalian cellular models is that they are often inherently different from those within intact tissue. For example, primary neuronal cultures do not achieve the functional and molecular compositional maturity of adult neurons^45,46^ and cell cultures do not contain the anatomical context of a tissue, particularly the diversity of cell types and physiological processes. Therefore, to investigate the molecular architecture of higher organisms, there is added value in preparing vitreous frozen tissue samples for cryoET.

### CryoCLEM of tissues

**Fig. 3:**
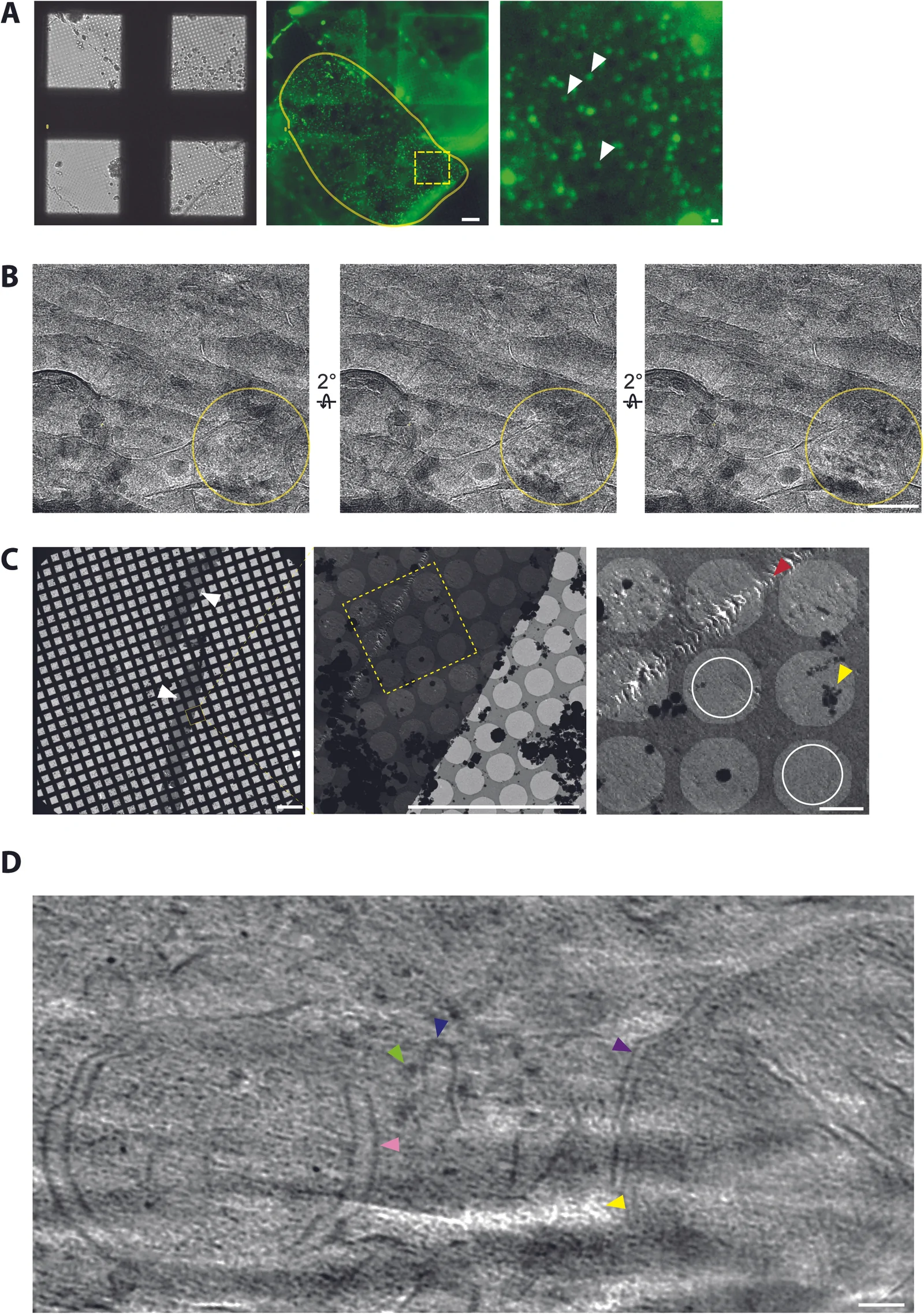
CryoCLEM and cryoET of tissue cryo-sections. (A) CryoFM of Psd95^GFP/GFP^ knockin mouse brain cortex cryo-sections on an EM grid. Left panel, brightfield image. Middle panel, GFP fluorescence. Yellow lariat, regions with good attachment of the tissue cryo-section to the EM grid indicated by GFP puncta in the same focal plane as the carbon foil. Scalebar, 20 μm. The dashed box shows the region enlarged in the right panel. Submicron sized puncta indicate the location of synapses within the tissue cryosection. Scalebar, 1 μm. (B) Electron micrographs showing partial devitrification of tissue cryo-sections. Each panel is related by a 2° tilt. Yellow circles, devitrified regions of ice evident from ice reflections appearing and disappearing from one tilt to the next. Scalebar, 100 nm. (C) Electron micrographs of cryo-section tissue ribbons from mouse brain. Left panel, low magnification montage of the EM grid showing tissue spanning multiple grid squares. Scalebar, 200 μm. Middle panel, medium magnification montage of grid squares on holey carbon foil. Scalebar, 20 μm. Yellow box, the region enlarged in the right panel. Yellow and red arrowheads indicate ice contamination and cutting damage from the knife, respectively. White circles, regions suitable for cryoET with minimal knife damage. Scalebar, 2 μm. (D) Tomographic slice of a mouse brain cryo-section showing in-tissue molecular architecture, including membranes, organelles and macromolecular complexes with light green, dark blue, pink, and purple arrowheads indicating a putative ribosome, the endoplasmic reticulum, an autophagosome intermediate, and a mitochondrion, respectively. Yellow arrowhead, low intensity voxels correspond to the tip of a ‘crevasse’ within the tissue caused by knife cutting damage. Scalebar, 50 nm.

To cryopreserve thicker tissue specimens, 100–200 μm acute tissue slices were prepared using a vibratome whilst maintaining viability by perfusing in carboxygenated physiological buffers (Fig. 3). Next, 2 mm biopsies of the tissue slice were collected and placed within a gold-coated carrier and vitrified by high-pressure freezing.^47^ This is a non-trivial step that required the use of iso-osmotic cryoprotectants, such as 20% dextran.^14^

To obtain samples suitable for cryoET, cryo-preserved tissue was sectioned by cryo-ultramicrotomy, also referred to as cryo-electron microscopy of thin vitreous sections (CEMOVIS).^14^ The carrier was trimmed around the target area of tissue leaving a trapezoid prism-shaped stub of tissue (60–100 × 100 × 60 μm), from which 70–150 nm thick cryo-sections were collected (Fig. 2) in a humidity-controlled environment (6–20% RH). Typically, sections were cut as a long ribbon containing multiple concatenated cryo-sections that were transferred and adhered by electrostatic charging on a holey carbon EM grid. In our cryoCLEM workflow, tissue sections were then imaged by cryoFM to map the location of our fluorescent protein. We also collected cryoFM Z-stacks to confirm that the tissue was in the same focal plane as the carbon foil, which gave an indication that cryo-sections were well attached (Fig. 3). Because cryo-sections were thinner than the focal depth of cryoFM, out of focus fluorescence was absent and sub-micron sized fluorescent puncta were detected within the tissue (Fig. 3).

It was also important to assess sample quality by cryoEM, particularly that the cryo-section was vitreous and had minimal knife damage (Fig. 3). De-vitrification can be detected by electron diffraction with an area aperture, but we found this was most efficiently tested by simply collecting images of the tissue at several tilt angles, which if devitrified will reveal ice reflections that appeared or disappeared (Fig. 3). Imaging the sample at high tilt also enabled us to confirm that the tissue was at the same focal plane as the carbon foil. This was important because tracking and focusing steps of batch cryoET data collection often failed if both the tissue cryo-section and carbon foil were not at eucentric height.

Cryo-tomograms reconstructed from cryo-sections revealed the in-tissue architecture of individual proteins (Fig. 3). However, these specimens also contained regions of knife damage including ruptures or ‘crevasses’ within the tissue and regions of compression^48^ (Fig. 3), which could be readily identified and excluded from further analyses.^49^ We suggest there is future potential for machine learning-based image restoration of knife damage, comparable to that developed for the missing wedge in cryotomograms.^50^ Despite providing the 3D molecular architecture of tissues, cryoCLEM and cryoET of cryo-sections is technically demanding.

**Fig. 4:**
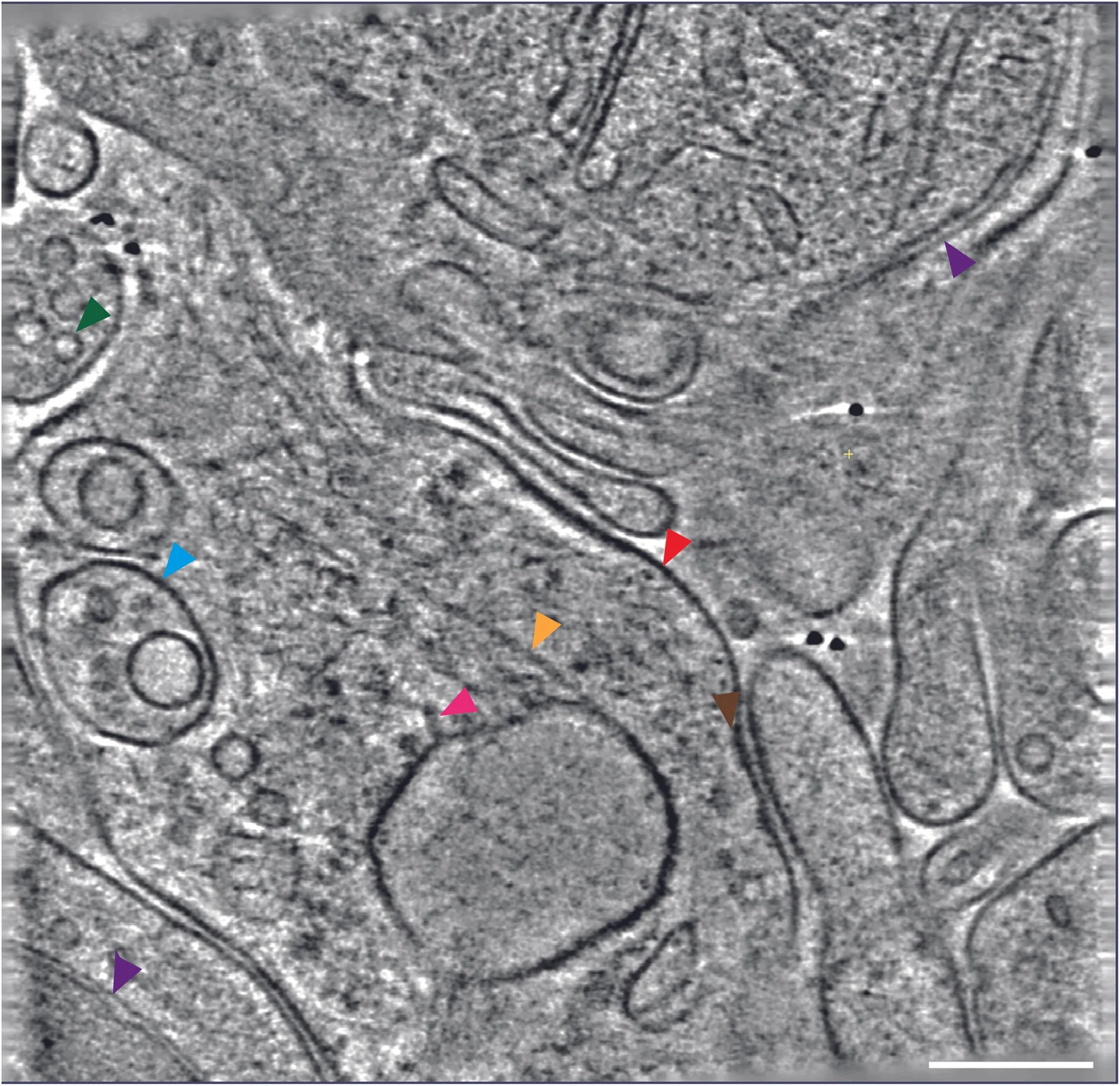
Molecular architecture within an ultra-fresh adult mouse forebrain sample by cryoET. The tomographic slice shows subcellular compartments containing organelles and macromolecular complexes, including: purple, green, light blue, gold, red, magenta, and brown arrowheads indicating a mitochondrion, a vesicle, a multivesicular body, the cytoskeleton, the plasma membrane, 25 nm membrane-associated protein, and a cluster of small proteins on the plasma membrane, respectively. Scalebar, 100 nm.

Therefore, we developed an ‘ultra-fresh’ method of preparing tissue for cryoCLEM and cryoET.^24^ Tissue or cell culture samples were homogenized in ice-cold physiological buffers, blotted and plunge frozen on EM grids. While cells were ruptured in these samples to produce fragments thin enough for cryoET, numerous subcellular compartments, including synapses, were left largely intact. Samples were prepared within two minutes at just above freezing temperature to inhibit enzyme-catalysed degradation, metabolic collapse, or other forms of deterioration that would otherwise unfold following homogenization. This approach can be targeted to particular anatomically-defined brain regions because each EM grid requires only ∼0.001% mouse forebrain. CryoFM detection of fluorescent labels within the sample was used to pinpoint the location of subcellular compartments within this complex mixture and direct the collection of cryoET data. Tomograms collected from ‘ultra-fresh’ samples revealed the molecular architecture, including the arrangement of individual macromolecular complexes, cytoskeletal elements and organelles (Fig. 4). This therefore represents the most straightforward approach for preparing tissue samples for cryoCLEM and cryoET experiments. While we have used cryoCLEM labels to investigate brain synapses,^24^ numerous other subcellular structures are evident in these tomographic data. Thus, we suggest many subcellular compartments with a smallest dimension less than the ice thickness (100–500 nm) of plunge-frozen EM grids should be amenable to this approach.

### Concluding remarks

We have discussed several workflows we have developed for integrating tissue sample preparation, cryoCLEM and cryoET with the aim of bridging length scales from molecules to the whole organism. We also demonstrated the use of genetically encoded fluorescent labels to target the collection of cryoET datasets from within tissues. For this there is the future promise of even greater positional accuracy using cryogenic super-resolution fluorescence microscopy. However, this is challenging because of the risk of devitrification caused by the high intensity illumination necessary for these techniques.^32,33^ Additional positional accuracy can also be achieved if the label itself is sufficiently electron dense to be observed directly within the cryoEM image. For this purpose, nanogold, quantum dots, or DNA origami scaffolds may be suitable.^51,52^ However, the size of these tags may be prohibitively large for accessing some targets, or may alter the native architecture.

The workflows we have developed for preparing tissue for cryoEM are complemented by new instrumentation for thinning *in vitro* cultured cell specimens using a focused ion beam scanning electron microscope (FIB-SEM) to mill the sample down to 200 nm lamellae for cryoET.^53,54^ For larger specimens of anatomically intact tissue, FIB-milling has recently been demonstrated in combination with cryo-lift out (cLO) to mill and transfer tissue lamellae onto EM grids.^55^ These procedures in principle provide higher quality samples for cryoET that lack knife damage, although cLO is also technically demanding and low throughput, and cryoCLEM workflows have yet to be developed with FIB-SEM/cLO. This and advances in instrumentation, automation and faster data collection strategies^56^ are therefore necessary to exploit the full potential of *in situ* structural biological methods.

It is our view that cryo-sectioning, FIB-milling, and ultra-fresh preparations are highly complementary. Cryo-sectioning, providing large (∼50× ∼10 000 μm) ribbons of tissue, enables rapid assessment of sample quality, including vitrification, and has the potential to obtain larger datasets of in-tissue tomograms, which complements the quality of cryoET from FIB-milled lamellae. While both cryo-sectioning and FIB-milling give a volume reconstruction within a 70–300 nm thick tissue sample, many subcellular structures are many times larger than this, including synapses and the cell nucleus, which are up to 3 μm and 6 μm diameter, respectively. Thus, a future advance in cryoET of tissue would be to register cryoCLEM serial cryo-sections (Fig. 2) to be able to reconstruct 3D volumes across multiple, aligned 150 nm thick tomograms.

## Ethical statement

Animals were treated in accordance with the UK Animal Scientific Procedures Act (1986) and NIH guidelines. Animal experiments were approved by the University of Leeds Animal Welfare and Ethics Committee.

## Author contributions

CL and CAL performed experiments. CAL and RAWF wrote and drafted the manuscript.

## Conflicts of interest

There are no conflicts of interest to declare.
